# About cancer screenings and saving lives: measuring the effects of cancer screening programs through meta-analyses—A comment to the meta-analysis “Estimated Lifetime Gained With Cancer Screening Tests” by Bretthauer et al. (2023)

**DOI:** 10.3389/fpubh.2024.1376377

**Published:** 2024-04-09

**Authors:** Fabrizio Stracci, Domenico Martinelli, Francesca Maria Anedda, Marta Caminiti, William Mantovani, Valentina Pettinicchio, Alessandra Sinopoli, Francesco Vitale, Roberta Siliquini, Walter Mazzucco

**Affiliations:** ^1^Section of Public Health, Department of Medicine and Surgery, University of Perugia, Perugia, Italy; ^2^Hygiene Unit, Department of Medical and Surgical Sciences, Policlinico Foggia Hospital, University of Foggia, Foggia, Italy; ^3^Screening Reference Center, Cagliari Local Health Unit, Cagliari, Italy; ^4^Coordinator of Organization and Evaluation Group, Italian Gruppo Screening del Cervicocarcinoma and Gruppo Screening ColoRettale, Firenze, Italy; ^5^School of Hygiene and Preventive Medicine, University of Perugia, Perugia, Italy; ^6^Clinical and Evaluative Epidemiology Unit, Local Health Trust, Trento, Italy; ^7^Screening Reference Center, Department of Prevention, Roma 1 Local Health Unit, Rome, Italy; ^8^Department of Prevention, Roma 1 Local Health Unit, Rome, Italy; ^9^Department of Health Promotion, Mother and Child Care, Internal Medicine and Medical Specialties, University of Palermo, Palermo, Italy; ^10^Department of Public Health and Pediatrics, University of Turin, Turin, Italy

**Keywords:** cancer, secondary prevention, screening programs, screening efficacy, cancer-specific mortality, all-cause mortality, lifetime gained, follow-up time

## 1 Introduction

A meta-analysis of randomized clinical trials conducted by Bretthauer et al. to evaluate the advantage of cancer screening, recently published by Jama Internal Medicine, concluded that “common cancer screenings do not save lives with the possible exception of sigmoidoscopy screening” (1). The Authors derive their conclusion from estimates of lifetime gained with screening by “comparing all-cause mortality in people who underwent screening with those who did not.” They used the relative risk of death from any cause measured from randomized trials of cancer screenings and the average follow-up time of the unscreened group to obtain estimates of lifetime gained with screening (1). Both Bretthauer et al. in their meta-analysis and a comment paper appeared in the same number of JAMA Internal Medicine express the view that only randomized controlled trials can provide evidence of (cancer) screening efficacy and that a reduction of all-cause mortality is the measure of choice to evaluate efficacy (instead of the commonly used cancer-specific mortality) (1, 2). The reason for their choice is that a reduced risk of cancer specific deaths, if it is not associated with a reduced risk of all-cause mortality, can be considered the consequence of deaths associated with harmful effects of screening counterbalancing the screening benefit or of substitution of cancer specific deaths with death from competing causes. Nevertheless, we contend that the use of too stringent criteria led to an underestimation of the influence of screening on all-cause mortality in the meta-analysis authored by Bretthauer et al. and that the use of all-cause mortality implies small and unreliable estimates of screening efficacy (1). We believe that small estimates of relative risk for all-cause mortality should not be interpreted as minor effect of a cancer screening but indicate the opportunity to investigate the presence of bias in cause of death assignment and eventual harm of screening. With respect to the results published by Bretthauer et al., we also remark that 10–15 years of follow-up are insufficient to fully evaluate the impact of screening. Furthermore, low adherence to screening and uptake of screening in the control arm led to underestimation of screening efficacy in some randomized trials. Finally, evidence from observational studies should not be completely ignored, particularly for cancer screening that reduces incidence of infiltrative cancers.

## 2 Cancer-specific vs. all-cause mortality to evaluate cancer screening efficacy

It is a matter of debate whether cancer-specific or all-cause mortality should be the principal measure to evaluate cancer screening efficacy (3–8). All-cause mortality provides a measure of screening impact on general mortality, which incorporates even treatment-related deaths and it is unbiased with respect to cause of death assignment (3). The choice of all-cause mortality as the main indicator for screening efficacy-a choice that can only be motivated by a theoretical reduced risk of bias-implies two important consequences. First, only a small reduction in all-cause mortality can be observed because an effective screening reduces cancer-specific deaths, which, in general, represent a small fraction of general mortality (2, 7). Similarly, all measures based on all-cause mortality including lifetime gained with screening in most cases lead to apparently limited or even trivial results because potentially important gains in a few individuals (i.e., cancer-specific deaths prevented with screening) are averaged for all screened (i.e., lifetime gained is calculated for the whole screened cohort). Screening is not proposed as the snake oil of western movies, which cures all disease, but to reduce mortality from a specific cancer. Indeed, the reason to use a screening test is that the target population contains a small fraction of people with asymptomatic disease or a pre-neoplastic condition, which can be detected as people testing positive and form a high-risk group. Thus, relatively few individuals can benefit from screening and it is unreasonable to expect a benefit of screening on the majority of the screened group without the target disease. Bretthauer and Kalager, which are among the Authors of the above meta-analysis, shared this view in a 2013 paper (9). In other words, the application of a screening test can neither lead to a dramatic change of all-cause mortality nor extend life expectancy of the whole screened cohort. Instead, it can result in an important mortality reduction in persons testing positive and even more, for people with screen-detected cancers or premalignant lesions (if overdiagnosis is not prominent among screen-detected lesions). As a motivated exception, low-dose CT reduced all-cause mortality in the National Lung Screening Trial (NLST) because the target population was at high risk and the fraction of deaths attributable to lung cancer was high (>20%) (10, 11). Thus, the definition of life saving intervention given above is puzzling if large all-cause mortality reductions and prolonged life duration are expected for large cohorts of subjects unaffected by the target disease. If we consider the statement: “life belt saves lives” (e.g., https://www.nhtsa.gov/seat-belts/seat-belts-save-lives), then would someone interpret its meaning as a claim of general protection against stroke, cancer and all other deaths or limited to road accidents? As in the case of specific cancer screenings, life belt can prevent deaths from car accident in a small fraction of drivers, which would have died if not wearing life belt in case of accident. Second, randomized trials of cancer screenings were designed to investigate a cancer-specific mortality reduction and, consequently, they cannot provide reliable estimates of all-cause mortality reduction due to insufficient power. For example, Stang and Jöckel showed that a relative reduction in breast cancer mortality of 20% can correspond to a reduction of maximum 1.7–1.8% all-cause mortality. Indeed, the assessment of an effective association between screening programs and all-causes mortality needs larger sample size than the one routinely adopted in the RCT (e.g., a population of about half a million is needed to demonstrate a significant 2–3% reduction of all-cause mortality at a 0.05 alpha level) (12). Actually, both supporters and opponents of all-cause mortality as the main measure of screening efficacy agree that available studies are too small to provide accurate estimates of all-cause mortality reduction for most cancer screenings (2, 11). Instead, screening programs targeted to high-risk populations for lethal cancers (e.g., lung cancer screening in former or actual smokers) and studies that combine testing for different cancers (e.g., the PLCO trial and possibly multi-cancer screening) require a smaller sample size (2, 10, 13).

Based on the above arguments, the finding of a small and uncertain impact of screening is not surprising in studies adopting all-cause mortality reduction or overall lifetime gained in the screened group as measures of health benefit. However, the Bretthauer's meta-analysis underestimated screening effects (1).

## 3 Follow-up time to assess all-cause mortality

Indeed, the Authors adopted a single 10- to 15-year follow-up time to assess all-cause mortality reduction for all different cancer screenings (1). They correctly acknowledged that this choice could have led to an underestimation of lifetime gained with screening. One of the references cited to justify the adopted follow-up threshold referred to a cancer-specific approach and suggested optimal follow-up times to evaluate all-cause mortality for different cancer screenings (14). Available evidence indicates that evaluation of cancer screenings impact on health outcomes often requires follow-up times longer than 15 years, and this may be particularly relevant for all-cause mortality assessment.

With respect to breast cancer, Heijnsdijk et al. stated that a significant all-cause mortality reduction can be detected only with a follow-up longer than 16 years (14). Also the investigators of the Canadian breast cancer screening trial reported changes in both mortality and overdiagnosis at 25-year follow-up and therefore recommended observation beyond 15 years (15, 16). More than a decade ago, the Swedish two-county trial reported 26-year follow-up results, showing significant breast-cancer specific mortality reduction. The study concluded recommending at least 20 years of follow-up to assess screening efficacy (17). Evidence from the ERSPC trial on prostate cancer screening showed that screening improved the risk of metastasis and disease-specific mortality at 21-year follow-up (18). The PLCO trial reported a small but significant reduction in all-cause mortality at the 17-year follow-up that was not present in the previous analysis with shorter follow-up (11 year) (13). Therefore, we believe that a too short follow-up was considered in the meta-analysis conducted by Bretthauer's et al. (1).

## 4 Other considerations

Moreover, incompleteness of cancer screenings included, and inconsistent list of selected cancer sites should be highlighted as limits of the analysis proposed by Bretthauer et al., despite the Authors' claim that they included in the analyses (a selection of) the most commonly used screening tests (1).

The worldwide diffused screenings, cervical cancer screening and colorectal cancer screening based on fecal immunochemical testing (FIT), were excluded because inclusion criteria were not met (availability of randomized controlled trial with ≥10-year follow-up). However, evidence of efficacy for cervical cancer screening from observational studies reporting a dramatic decrease of invasive cervical cancer is considered sufficient (19, 20). Indeed, cervical cancer screening has the potential to contribute to lifetime gains because deaths averted occur on average at a younger age if compared to many other screened cancers (21). Furthermore, in certain cases, the notion that the efficacy of a new screening test can only be measured by a randomized controlled trial, which require very a large sample size and long-term follow-up, makes the evaluation of a new test almost impractical. In fact, for ethical reasons, a new test (e.g., tomosynthesis vs. digital mammography in breast cancer screening, immunochemical vs. guaiac stool test) must be compared with the established older test. Thus, the omission of cervical cancer screening and FIT-based large bowel screening, though in agreement with the study inclusion criteria, reduced the validity of the Authors' general statement that cancer screenings does not save lives.

Furthermore, even if “commonly used” is not a delimitating definition, it is difficult to include lung cancer screening under that heading (22, 23).

Besides providing estimates based on too short follow-up times if compared to evidence from the literature and excluding screening with evidence of efficacy from observational studies, Bretthauer et al. also disregarded the problems of “non-participation” and “contamination” (i.e., diffusion of screening in the control group), which both can determine an underestimation of screening efficacy. Participation varies by cancer screening and randomized trial (e.g., it was as low as 40% in the colonoscopy trial and 58% in the Italian sigmoidoscopy trial) (24, 25). Screening in the control group was frequent in some of the meta-analyzed trials (e.g., substantial use of PSA testing in the PLCO trial and 47% endoscopy in the US sigmoidoscopy trial) (26). Then the proposed figures for lifetime gains obtained with screenings may be underestimated and per-protocol estimates should have been reported for comparison (27).

## 5 Conclusion

In conclusion, the statement that cancer screenings do not save lives cannot be properly drawn from the Bretthauer's et al. meta-analysis because lifetime gains are likely underestimated and based on uncertain all-cause mortality estimates (1). Instead, we can agree with Welch et al. that randomized trials of new screening tests, including multi-cancer tests, are necessary before widespread population use (2). Furthermore, we believe that all-cause mortality reduction should be considered a complementary measure to assess the presence of bias in randomized trials of cancer screening or in meta-analysis of these studies, and that the use of this indicator should be limited to studies with adequate sample size. Lifetime gains estimated for the screened group from all-cause mortality reduction is a misleading measure and should be avoided because it implies a benefit for all persons in the screening group, including those not affected by the target cancer. Of interest, meta-analyses of observational studies dealing with cancer screenings should be considered as well to provide a clear and complete picture on their effectiveness (28, 29).

Lastly, studies of cancer screening providing health outcomes estimates in term of QALY would be important, as they could incorporate both health benefits, like cancer-specific mortality reduction, and harms of screening (e.g., overdiagnosis) in a single indicator.

## Author contributions

FS: Conceptualization, Methodology, Writing—original draft, Writing—review & editing. DM: Methodology, Writing—original draft, Writing—review & editing. FMA: Supervision, Writing—review & editing. MC: Methodology, Supervision, Writing—review & editing. WMan: Supervision, Writing—review & editing. VP: Supervision, Writing—review & editing. AS: Supervision, Writing—review & editing. FV: Conceptualization, Supervision, Writing—review & editing. RS: Supervision, Writing—review & editing. WMaz: Conceptualization, Methodology, Writing—review & editing.
